# Efficacy and tolerability of preoperative chemoradiotherapy with S-1 alone for locally advanced rectal cancer

**DOI:** 10.1093/jrr/rraa117

**Published:** 2020-12-21

**Authors:** Nobuki Imano, Yuji Murakami, Katsumaro Kubo, Daisuke Kawahara, Yuki Takeuchi, Ikuno Nishibuchi, Tomoki Kimura, Masatoshi Kochi, Yuji Takakura, Wataru Shimizu, Hiroyuki Egi, Shinnosuke Uegami, Hiroki Ohge, Shinya Takahashi, Hideki Ohdan, Yasushi Nagata

**Affiliations:** Department of Radiation Oncology, Graduate School of Biomedical Health Sciences, Hiroshima University. Hiroshima, Japan; Department of Radiation Oncology, Graduate School of Biomedical Health Sciences, Hiroshima University. Hiroshima, Japan; Department of Radiation Oncology, Graduate School of Biomedical Health Sciences, Hiroshima University. Hiroshima, Japan; Department of Radiation Oncology, Graduate School of Biomedical Health Sciences, Hiroshima University. Hiroshima, Japan; Department of Radiation Oncology, Graduate School of Biomedical Health Sciences, Hiroshima University. Hiroshima, Japan; Department of Radiation Oncology, Graduate School of Biomedical Health Sciences, Hiroshima University. Hiroshima, Japan; Department of Radiation Oncology, Graduate School of Biomedical Health Sciences, Hiroshima University. Hiroshima, Japan; Department of Gastroenterological and Transplant Surgery, Graduate School of Biomedical and Health Sciences, Hiroshima University. Hiroshima, Japan; Department of Gastroenterological and Transplant Surgery, Graduate School of Biomedical and Health Sciences, Hiroshima University. Hiroshima, Japan; Department of Gastroenterological and Transplant Surgery, Graduate School of Biomedical and Health Sciences, Hiroshima University. Hiroshima, Japan; Department of Gastroenterological and Transplant Surgery, Graduate School of Biomedical and Health Sciences, Hiroshima University. Hiroshima, Japan; Department of Surgery, Graduate School of Biomedical and Health Sciences, Hiroshima University. Hiroshima, Japan; Department of Surgery, Graduate School of Biomedical and Health Sciences, Hiroshima University. Hiroshima, Japan; Department of Surgery, Graduate School of Biomedical and Health Sciences, Hiroshima University. Hiroshima, Japan; Department of Gastroenterological and Transplant Surgery, Graduate School of Biomedical and Health Sciences, Hiroshima University. Hiroshima, Japan; Department of Radiation Oncology, Graduate School of Biomedical Health Sciences, Hiroshima University. Hiroshima, Japan

**Keywords:** locally advanced rectal cancer, preoperative chemoradiotherapy, S-1 alone

## Abstract

Preoperative chemoradiotherapy with capecitabine or 5-fluorouracil is a standard treatment for locally advanced rectal cancer (LARC). S-1, a prodrug of 5-fluorouracil, is a candidate for this chemoradiotherapy regimen in Japan; however, treatment outcomes after S-1 treatment alone are not clear. This study aimed to assess the efficacy and tolerability of preoperative chemoradiotherapy with S-1 alone for LARC. We retrospectively evaluated 54 LARC patients who underwent preoperative chemoradiotherapy with S-1 alone in our institution between 2005 and 2017. The clinical tumor stage was cT2–3 in 31 patients and cT4 in 23 patients, and lymph node metastases were clinically evident in 31 patients. S-1, at a dose of 80 mg/m^2^/day, was orally administered during radiotherapy. A total dose of 45–50.4 Gy was delivered in 25–28 fractions (median: 50.4 Gy). Surgical resections were scheduled 6–10 weeks after chemoradiotherapy completion. The 3- and 5-year overall survival rates were 92.4 and 72.8%, respectively, with a median follow-up time of 51 months. The 3- and 5-year local control rates were 96.2 and 85.9%, respectively. A pathological complete response was observed in 7 patients (13.0%) at the time of surgery. Ten patients (18.5%) had grade 3 acute toxicities and 5 patients (9.3%) had grade 3 late toxicities. No grade 4 or 5 toxicities were observed. Preoperative chemoradiotherapy with S-1 alone followed by total mesorectal excision resulted in a low incidence of toxicities and comparable clinical results. Therefore, S-1 alone can be a treatment option for preoperative chemoradiotherapy in LARC patients.

## INTRODUCTION

Preoperative chemoradiotherapy (CRT) is a standard treatment for locally advanced rectal cancer (LARC) [1, 2]. It has been reported that preoperative CRT for LARC improves local control and anal preservation rates [1–4]. Another preoperative treatment option for LARC is short-course radiotherapy (RT) [5, 6]. Although a clear advantage has not been shown, long-course CRT is currently the recommendation in several guidelines [7–9], especially in advanced cases such as clinical tumor stage T4 and positive lymph node metastases, due to concerns about efficacy and late toxicities. For long-course RT, capecitabine or 5-fluorouracil (5-FU) has been adopted as the standard concurrent chemotherapy regimen [1–6, 10–14]. S-1, a prodrug of 5-FU, is one of the candidates for this chemotherapy regimen in Japan [15–25]. There are some studies of S-1 in combination with other drugs [15–19] and S-1 alone [20–24] for preoperative CRT for LARC. For preoperative CRT with S-1 alone, fewer adverse events have been reported, with a similar efficacy compared to that with capecitabine [23]. S-1 alone could be a useful option for preoperative CRT for LARC. However, there are very few reports on the treatment outcomes of preoperative CRT with S-1 alone for LARC, especially regarding long-term results [25], and the number of patients in these studies was limited [20–25]. In this study, we retrospectively assessed the efficacy and tolerability of S-1 in preoperative CRT for LARC. In addition, there are very few reports examining the appropriateness of irradiation field size and dose fractionation, although there are many studies on concomitant chemotherapy [10–24]. In this study, we also examined the field size and dose fractionation in RT with S-1 alone.

**Fig. 1.**
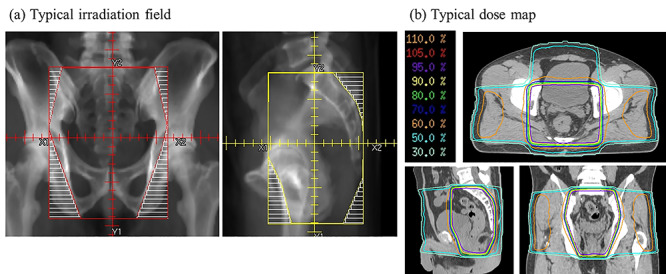
(**a**) Typical irradiation field, (**b**) typical dose map.

## MATERIALS AND METHODS

### Eligibility criteria

Patients were considered eligible for this retrospective analysis if they had histologically confirmed rectal cancer, had been diagnosed as clinical stage II or III according to the 7th TNM staging system of the International Union Against Cancer (UICC), had tumor at or below peritoneal reflection, and had received preoperative CRT with oral S-1 between 2005 and 2017 at our institution. Clinical stages were assessed using physical examination findings and computed tomography (CT). Magnetic resonance imaging (MRI) and ^18^F-fluorodeoxyglucose positron emission tomography/CT (18F-FDG PET/CT) findings were referred to, when they were available. In total, 54 patients were included in this study. This retrospective analysis was approved by the institutional review board.

### Radiotherapy

CT (2.5 mm slice) was conducted for RT planning. The target volumes and normal tissue structures were contoured on each axial CT slice. Gross tumor volume (GTV) was contoured with reference to MRI and PET/CT images. Lymph node metastases were considered as positive if the shortest axial diameter was ≥10 mm on CT or MRI images or if there was abnormal focal 18F-FDG uptake on PET/CT. The clinical target volume (CTV) included the primary GTV with a 3-cm margin in the longitudinal direction and a 5-mm margin in the other directions, the lymph node metastases, the mesorectum and regional lymph nodes (the internal iliac, obturator and presacral lymph nodes up to L5/S1). In the case of T4 disease invading the bladder, prostate, uterus or vagina, it was decided to include the external iliac lymph node area as CTV. 3D RT treatment planning was performed with 6–18 MV X-rays, using the 3- or 4-field technique. A typical irradiation field and dose map are shown in Fig. 1.The 7 initial patients, treated before April 2008, were administered a total dose of 45 Gy in 25 fractions. The subsequent 5 patients, treated before December 2012, were treated with 50 Gy in 25 fractions. One patient was treated with 46 Gy in 23 fractions in this period. Beginning in January 2012, a dose of 50.4 Gy in 28 fractions was adopted for the remaining 41 cases.

## Chemotherapy

All patients received concurrent chemotherapy with oral S-1. Specifically, S-1 at a dose of 80 mg/m^2^/day was orally administered twice a day on days 1–14 and 22–35, concomitantly with RT. The dosages of chemotherapeutic agents were reduced by 20–50% when necessary, according to the patient’s age, renal function and general condition.

## Surgery

Total mesorectal excision was scheduled 6–10 weeks after the completion of CRT. Unilateral or bilateral lateral lymph node dissections (LLNDs) were performed in patients who were diagnosed with lateral lymph node (LLN) metastases by the initial imaging examinations. The tumor responses to neoadjuvant CRT were evaluated by histopathological examination.

### Follow-up

Patients were followed up at 1 and 3 months after treatment completion, and every 3 months thereafter. The patients’ treatment responses and failures were evaluated using a chest-to-pelvis CT at the 6-month follow-up visit and every 6–12 months thereafter. Pelvic MRI or PET/CT was performed when necessary. Colonoscopy was performed every year until 3 years after surgery and biopsies were performed when recurrence was suspected. Acute toxicity was defined as a toxic event that occurred between the initiation of RT and surgery. Late toxicity was defined as a toxic event that occurred or persisted beyond 3 months after surgery. For toxicities, the Common Terminology Criteria for Adverse Events (CTCAE) version 4.0 was used.

### Statistical analysis

Overall survival (OS) was defined as the period from the initiation of treatment to death from any cause. Progression-free survival (PFS) was defined as the time from the initiation of treatment to the progression of LARC or death from any cause. Local control rate (LCR) was defined as the percentage of patients without pelvic cavity recurrence. The Kaplan–Meier method was used to calculate survival and control rates. For univariate analysis (UVA), the log rank test was used to compare survival rates or LCR between two groups and the Fisher’s exact test was used to compare pathological complete response (pCR) rates and the incidence of toxicities between two groups. For multivariate analysis (MVA), the Cox regression model was used to analyze survival rates. Statistical analyses were performed using JMP Pro 14 (SAS Institute Inc., Cary, NC, USA).

## RESULTS

### Patient and tumor characteristics

The patient and tumor characteristics are summarized in Table 1. There were 41 men and 13 women. The median age was 63 years (range, 30–85 years). All patients had adenocarcinoma. The degree of differentiation was as follows: well-differentiated adenocarcinoma in 23 patients, moderately differentiated adenocarcinoma in 28 patients and poorly differentiated adenocarcinoma in 3 patients. The clinical tumor stage was cT2 in 5 patients (all had lymph node metastases), cT3 in 26 patients, cT4a in 13 patients and cT4b in 10 patients. Additionally, 31 patients had clinical evidence of lymph node metastases. There were 18 patients with cStage II disease and 38 patients with cStage III disease.

**Table 1:** Patient and tumor characteristics

Age (years)	Median (range)	63 (30–85)
Sex	Male/female	41/13
PS	0/1/2	46/7/1
Double cancer	Yes/no	8/46
Hemoglobin	Median (range)	13.2 (7.5–18.2)
CEA	Median (range)	5.8 (1.0–276.9)
CA19–9	Median (range)	15 (< 2.0–104)
Tumor site	Rb/Rab/Ra	29/13/12
Pathology	Adenocarcinoma (well/mod/por)	54 (23/28/3)
T factor	1/2/3/4	0/5/26/23
N factor	0/1/2	21/20/13
Stage	II/III	21/33

well = well-differentiated tubular adenocarcinoma, mod = moderately differentiated tubular adenocarcinoma, por = poorly differentiated adenocarcinoma, PS = performance status, CEA = carcinoembryonic antigen, CA 19–9 = carbohydrate antigen 19–9.

History of double cancer was observed in 8 patients with 10 malignancies. These malignancies were distributed as follows: 3 patients had colon cancer, and each of 7 patients had the following: thyroid cancer, lung cancer, gastric cancer, renal cancer, prostate cancer, lymphoma and meningioma.

### Treatment

Treatment methods are summarized in Table 2. The median total irradiation dose was 50.4 Gy in 28 fractions (range, 45–50.4 Gy in 25–28 fractions). The completion rate of planned RT was 98.1% (53/54). In 1 patient, RT was discontinued early, at a dose of 46.8 Gy, due to grade 3 diarrhea. CRT interruption was required in 2 patients: 1 patient, due to abdominal abscess and 1 patient, due to appendicitis. The total irradiated dose was ≥50 Gy in 45 cases (83%) and <50 Gy in 9 cases (17%). In 6 patients (11%), S-1 was reduced by 20–50% from the start of treatment according to the patient’s age (3 patients), renal function (2 patients) and general condition (1 patient). The completion rate of oral S-1 administration was 88.9% (48/54). In 6 patients, S-1 was discontinued due to acute toxicities (grade 3 neutropenia in 2 patients, grade 2 anorexia in 2 patients, grade 3 anemia in 1 patient and grade 3 diarrhea in 1 patient). Surgical resection was performed during 6–10 weeks following the completion of CRT (median: 57 days) in principle. In 4 patients, resections were performed not at initial schedules but at the point when tumor regrowths were found (140, 175, 195 and 278 days, respectively). Surgical procedures are shown in Table 2c. LLNDs were performed in 16 patients initially diagnosed with LLN metastases.

**Table 2:** Treatment method

(a) Radiotherapy		
Field	3-field	4
	4-field	50
Prescribed dose	50.4 Gy/28 fractions	41
	45 Gy/25 fractions	7
	50 Gy/25 fractions	5
	46 Gy/23 fractions	1

(b) Chemotherapy		
Administration	Days 1–14, 22–35	43
	On RT day	11
(c) Surgery		
Time from CRT (days)	Median (range)	57 (7–278)
Surgical procedure	Abdominoperineal resection	23
	Low anterior resection	22
	Intersphincteric resection	5
	Hartmann’s operation	2
	Total pelvic exenteration	1
Surgical approach	Open	20
	Laparoscopic	34
Lateral lymph node dissection	Yes	16
	No	38


### Efficacy of chemoradiotherapy with S-1 alone

Seven patients (13%) achieved pCR and 44 patients (81.5%) achieved pathological downstaging. R0 resection was achieved in all patients. The predictive factors for pCR are summarized in Table 3. The UVA showed that T stage was associated with pCR. Additionally, pCR was observed in 7 patients (22.6%) with T2–3 disease, whereas pCR was not observed in any patients with T4 disease. (T2–3 vs T4: 22.6 vs 0%, *P* = 0.016).

**Table 3:** Predictive factors of pathological complete response

			UVA	
Factors		*n*	pCR	*P* value
Age (years)	<63	20	2 (10.0%)	1
	≥63	34	5 (14.7%)	
Sex	Male	41	4 (9.8%)	0.34
	Female	13	3 (13.0%)	
T stage	T2/T3	31	7 (22.6%)	0.016^*^
	T4	23	0 (0%)	
N stage	N0	21	2 (9.5%)	0.693
	N1/N2	33	5 (15.2%)	
UICC stage	Stage II	21	2 (9.5%)	0.693
	Stage III	33	5 (15.2%)	
Differentiation	Well	23	3 (13.0%)	1
	Moderate/poor	31	4 (12.9%)	
Hemoglobin	<11.0 g/dl	12	1 (8.3%)	1
	≥11.0 g/dl	42	6 (14.3%)	
CEA	<15.0 ng/ml	39	7 (18.0%)	0.171
	≥15.0 ng/ml	15	0 (0%)	
CA19–9	<37.0 U/ml	46	7 (15.2%)	0.577
	≥37.0 U/ml	8	0 (0%)	
Sphincter preservation	Yes	27	2 (7.4%)	0.42
	No	27	5 (15.5%)	
Lateral lymph node dissection	Yes	16	6 (15.8%)	0.66
	No	38	1 (6.3%)	
Total dose	<50 Gy	9	0 (0%)	0.586
	≥50 Gy	45	7 (15.6%)	
Postoperative chemotherapy	Yes	37	3 (8.1%)	0.189
	No	17	4 (23.5%)	

^*^ *P* < 0.05. UV =, univariate analysis, CEA = carcinoembryonic antigen, CA19–9 = carbohydrate antigen 19–9.

### Survival rate and patterns of failure

**Fig. 2.**
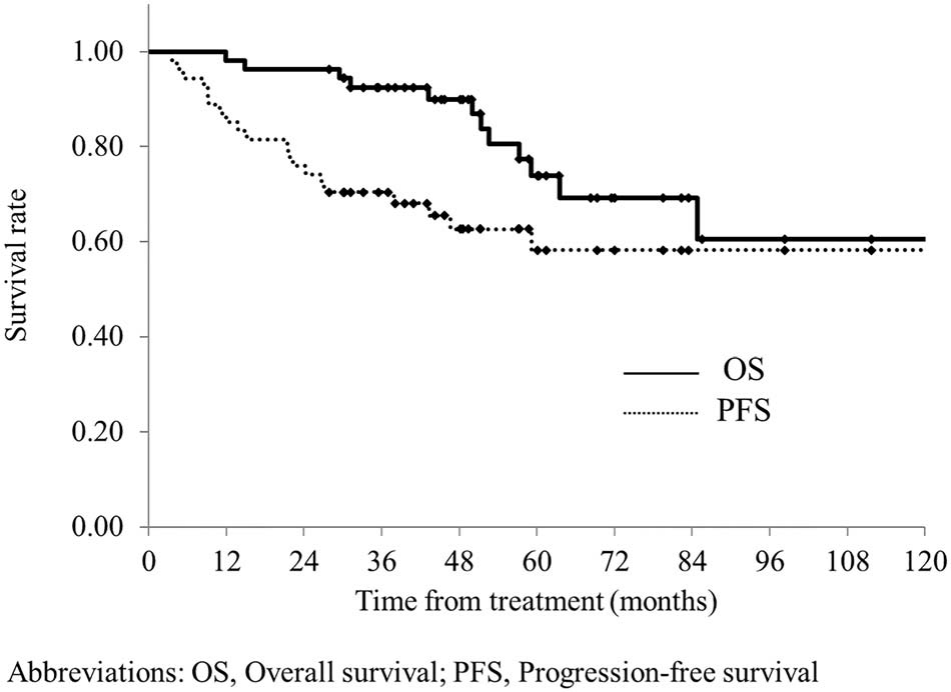
Overall survival and progression-free survival for all patients. The median follow-up period was 51 months (range, 12–155 months) for all patients and 56 months (range, 28–155 months) for survivors. The 3- and 5-year OS rates were 92.4 and 72.8%, respectively. The 3- and 5-year PFS rates were 70.4 and 58.1%, respectively.

**Fig. 3.**
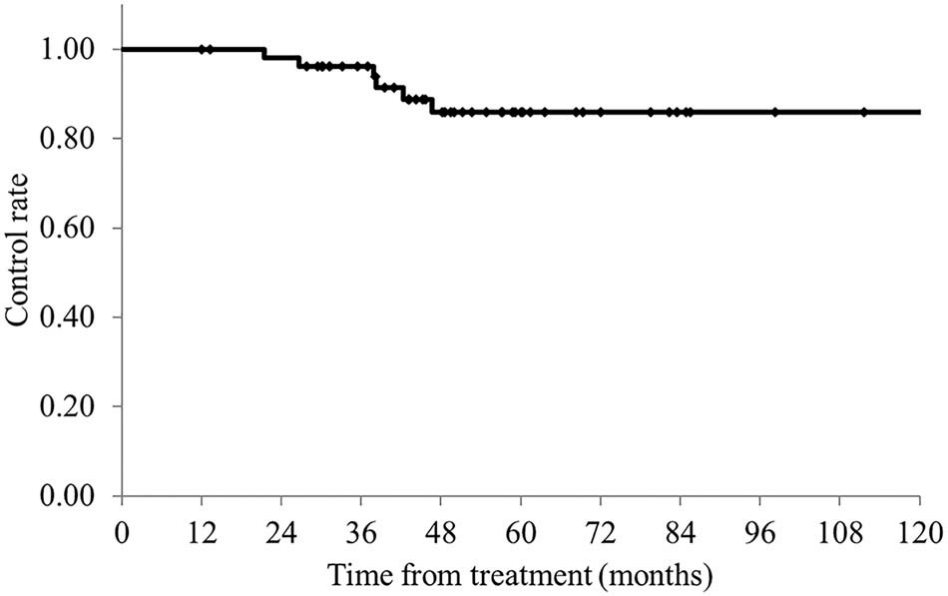
Local control rates for all patients. The 3- and 5-year LCRs were 96.2 and 85.9%, respectively.

The median follow-up period was 51 months (range, 12–155 months) for all patients and 56 months (range, 28–155 months) for survivors. The 3- and 5-year OS rates were 92.4 and 72.8%, respectively (Fig. 2); the 3- and 5-year PFS rates were 70.4 and 58.1%, respectively; and the 3- and 5-year LCRs were 96.2 and 85.9%, respectively (Fig. 3). Recurrence was observed in 19 cases. The patterns of initial failures included the following: local failure in 3 patients, local and distant (lung) failure in 1, and distant failure in 15 patients (9 in the lung, 4 in the liver and 2 in a distant lymph node). Local failure was observed in 2 cases after distant failure, and a total of 6 local recurrences were observed (5 failures involving the primary lesion and 1 pelvic lymph node failure). No recurrence was observed in the marginal region of the irradiation field. The prognostic factors for OS and PFS are summarized in Table 4. Both the UVA and MVA showed that the degree of pathological differentiation and carcinoembryonic antigen (CEA) levels were associated with OS. CEA level was the only factor associated with PFS after UVA. Nevertheless, no factors were related to LCR: patients treated with 50–50.4 Gy have a tendency to have better LCR compared with those treated with 45–46 Gy (5-year LCR, 50–50.4 Gy vs 45–46.8 Gy: 89.6 vs 68.6%, *P* = 0.26).

### Toxicities

Table 5a shows the acute toxicities observed. Regarding the hematological toxicities, grade 3 toxicities included anemia in 2 cases (3.7%) and leukopenia in 2 cases (3.7%). With respect to non-hematological toxicities, grade 3 toxicities included diarrhea in 3 patients (5.6%), mucositis in 1 patient (1.9%), perirectal abscess in 1 patient (1.9%) and appendicitis in 1 patient (1.9%). In total, 10 of 54 patients (18.5%) had grade 3 toxicities, and no grade 4 or 5 acute toxicities were observed. There was no difference in the incidence of grade 3 acute toxicities between 47 patients whose planed dose was 50.4 Gy/28 fractions or 50 Gy/25 fractions and 7 patients whose planed dose was 45 Gy/25 fractions or 46 Gy/23 fractions (19.6 vs 12.5%, *P* = 1.00).

**Table 4:** Prognostic factors of OS and PFS

			UVA for OS		MVA for OS		UVA for PFS	
Factors		*n*	5-year OS	*P* value	HR	*P* value	5-year PFS	*P* value
			(95% CI)		(95% CI)		(95% CI)	
Age (years)	≤63	28	77.4% (55.4–90.4%)	0.186	-	-	60.3% (41.4–76.5%)	0.82
	>63	26	58.4% (30.7–81.7%)				50.1% (24.5–75.7%)	
Sex	Male	41	78.0% (57.8–90.2%)	0.409	-	-	52.9% (34.2–70.8%)	0.504
	Female	13	63.5% (32.9–86.0%)				69.2% (40.9–88.0%)	
T stage	T2/T3	31	80.8% (60.7–92.0%)	0.693	-	-	71.0% (53.0–84.1%)	0.213
	T4	23	61.8% (33.1–84.1%)				32.8% (10.8–66.3%)	
N stage	N0	21	73.2% (45.6–89.9%)	0.667	-	-	57.1% (33.8–77.7%)	0.904
	N1/N2	33	73.8% (51.2–88.4%)				57.8% (36.8–76.3%)	
UICC stage	Stage II	21	73.2% (45.6–89.9%)	0.667	-	-	57.1% (33.8–77.7%)	0.904
	Stage III	33	73.8% (51.2–88.4%)				57.8% (36.8–76.3%)	
Differentiation	Well	23	90.9% (56.1–98.7%)	0.036^*^	8.85 (1.06–73.76)	0.044^*^	73.9% (52.8–87.8%)	0.257
	Moderate/poor	31	65.1% (44.8–81.0%)				50.1% (31.5–68.7%)	
Hemoglobin	<11.0 g/dl	12	40.7% (20.7–78.6%)	0.222	-	-	44.4% (13.7–80.0%)	0.651
	≥11.0 g/dl	42	81.7% (64.0–91.8%)				61.7% (45.3–75.8%)	
CEA	<15.0 ng/ml	39	84.1% (66.6–93.3%)	0.010^*^	6.07 (1.48–24.96)	0.012^*^	67.0% (50.0–80.4%)	0.047^*^
	≥15.0 ng/ml	15	21.7% (3.0–71.0%)				0%	
CA19–9	<37.0 U/ml	46	72.5% (54.3–85.4%)	0.82	-	-	55.5% (38.8–71.1%)	0.507
	≥37.0 U/ml	8	58.3% (15.7–91.3%)				75.0% (37.7–93.7%)	
Sphincter preservation	Yes	27	82.3% (60.7–93.4%)	0.678	-	-	64.7% (44.5–80.8%)	0.506
	No	27	63.2% (37.2–83.3%)				50.0% (27.2–72.8%)	
Lateral lymph node dissection	Yes	16	73.2% (49.9–88.3%)	0.776	-	-	54.7% (30.4–77.0%)	0.445
	No	38	72.9% (45.8–89.5%)				57.3% (36.4–75.8%)	
Total dose	<50 Gy	9	44.4% (17.7–74.9%)	0.104	-	-	44.4% (17.7–74.9%)	0.578
	≥50 Gy	45	84.8% (67.0–93.9%)				66.0% (50.9–78.4%)	
Postoperative chemotherapy	Yes	37	75.8% (45.2–92.2%)	0.884	-	-	54.8% (36.5–71.9%)	0.491
	No	17	73.9% (53.3–87.5%)				66.9% (39.7–87.5%)	

^*^ *P* < 0.05. HR = hazard ratio, CI = confidence interval, CEA = carcinoembryonic antigen, CA19–9 = carbohydrate antigen 19–9.

**Table 5:** Toxicities

(a) Acute toxicities		
	G 2	G 3
Diarrhea	13 (24.1%)	3 (5.6%)
Mucositis	21 (38.9%)	1 (1.9%)
Perirectal abscess		1 (1.9%)
Appendicitis		1 (1.9%)
Dermatitis	5 (9.2%)	
Anorexia	2 (3.7%)	
Neutropenia	5 (9.2%)	2 (3.7%)
Anemia	4 (7.4%)	2 (3.7%)
Hyperbilirubinemia	1 (1.9%)	
All toxicities	25 (46.3%)	10 (18.5%)
(b) Late toxicities		
	G 2	G 3
Ileus	1 (1.9%)	1 (1.9%)
Intestinal stenosis		1 (1.9%)
Abdominal abscess		1 (1.9%)
Rectovesical fistula		1 (1.9%)
Ureteral fistula		1 (1.9%)
Ureteral obstruction	1 (1.9%)	
Sacral fracture	1 (1.9%)	
All toxicities	3 (5.6%)	5 (9.2%)

Late toxicities are shown in Table 5b. The grade 3 toxicities were as follows, each affecting one patient: ileus (1.9%), intestinal stenosis (1.9%), abdominal abscess (1.9%), rectovesical fistula (1.9%) and ureteral fistula (1.9%). In total, 5 patients (9.3%) had grade 3 late toxicities. No grade 4 or 5 late toxicities were observed. Two of the 7 patients (28.5%) prescribed 45 Gy had grade 3 late toxicities, and 3 of 47 (6.4%) patients prescribed 50 or 50.4 Gy had grade 3 late toxicities. The 45 Gy group tended to have more late toxicities, but at least there was no increase in late toxicities with increasing irradiation dose.

## DISCUSSION

Preoperative CRT is the standard treatment for LARC [1, 2]. Various treatment strategies have been developed for RT, chemotherapy and surgery to improve treatment results [1–6, 10–24]. At present, the most common method is preoperative CRT with 45–50.4 Gy/25–28 fractions with capecitabine or 5-FU, followed by surgical resection 6–8 weeks after CRT [7–9]. The results of previous randomized controlled trials (RCT) showed that preoperative CRT yielded a pCR rate of 10–20%, 3- and 5-year OS rates of 75–88 and 65–80%, respectively, and 3- and 5-year PFS rates of 65–76 and 55–68%, respectively [1–6, 10–14]. The rates of acute and late toxicities during CRT are reported to be about 8–27% and 7–25%, respectively [1–6, 10–14]. In our study, we performed RT primarily with 50.4 Gy/28 fractions in combination with S-1 alone, and our study showed pCR of 13%, 3- and 5-year OS rates of 92.4 and 72.8%, respectively, and 3- and 5-year PFS rates of 70.4 and 58.1%, respectively. These outcomes were similar to the previous RCT results. Since >40% of patients in our study had T4, our results seemed favorable considering that >80–90% of the cases included in the RCTs involved T1–T3 disease. Additionally, in our study, the rates of acute and late toxicities were 18.6 and 9.3%, respectively, which were also comparable to those reported by previous RCTs [1–6, 10–14]. Regarding LCRs, the 5-year LCR was reported as 90–95% in previous RCTs [1–6, 10–14], making our finding of 85.9% slightly inferior to those of previous reports. This may be due to the higher number of T4 cases in our study than in the previous studies. In fact, of the 6 cases involving local recurrences in our study, 5 involved recurrences around the primary lesion, and 4 of the 5 cases involved T4 disease. One patient had a recurrence in the LLN. The LLN metastases was detected by CT image and LLND was performed in this patient. Although LLND was omitted in patients without LLN metastases in our study, none of these patients had a recurrence in the LLN region. Therefore, LLND could be omitted in patients without LLN metastases who were managed using CRT with S-1 alone.

According to the results of past RCTs [1–6, 10–14] and several guidelines [7–9], capecitabine and 5-FU are considered as the standard agents for chemotherapy combined with preoperative radiation therapy for LARC. Combinations of oxaliplatin, irinotecan and other drugs have been validated, but none have shown clear superiority [10–14]. Similar effects were shown for capecitabine and 5-FU, but 5-FU was reported to have fewer adverse events [14]. S-1, a prodrug of 5-FU, is one of the candidates for this chemotherapy regimen in Japan. There are several reports on S-1 alone [20–25] and combined therapy with other chemotherapeutic agents [15–19] for preoperative CRT for LARC, but there are few reports showing the treatment outcomes of S-1 alone, especially with regard to long-term results [25]. Table 6 shows the results of CRT using S-1 alone. The downstaging and pCR rates in our study are comparable to those in previous studies using S-1 alone [20–25]. Although only one study, by Hiratsuka *et al*. [25], showed survival rates, our results were similar to theirs, and both are comparable to those of an RCT conducted previously [1–6, 10–14]. In terms of adverse events, 18.5% were grade 3 acute toxicities in our study. In the 6 studies on S-1 administration alone (including our study), the rates of grade 3 acute toxicities were reported to be 0–22% [20–25] (Table 6). These results are also equal to or less than the 8–40% rate found in past RCTs [1–6, 10–14]. One report compares the effects and toxicities of capecitabine and S-1 as regimens in preoperative CRT for LARC. Although the effects were similar in both groups, there were fewer adverse events in the S-1 group [22]. Considering these results, S-1 alone may be a useful option in terms of both efficacy and safety as a chemotherapeutic regimen in preoperative CRT for LARC.

**Table 6:** Reports on preoperative chemoradiotherapy for locally advanced rectal cancer using S-1 alone.

	Year	*n*	cT4	cN+	RT	S-1	S-1 dose	Acute toxicity ≤Grade 3	pCR	Down staging	5-year OS
Sadahiro *et al*. [20]	2011	30	8 (26.7%)	22 (73.3%)	45 Gy/25fx	Day 1–14, 22–35	65–80 mg/m^2^	6 (22.2%)	6 (22.2%)	21 (77.8%)	-
Morimoto *et al*. [21]	2012	9	-	-	40 Gy/20fx	On RT day	80–100 mg/m^2^	2 (22.2%)	0 (0%)	PR: 5 (55.6%)	-
Funahashi *et al*. [22]	2014	9	0 (0%)	5 (55.6%)	45 Gy/25fx	On RT day	65–80 mg/m^2^	0 (0%)	1 (11.1%)	-	-
Su *et al*. [23)	2014	24	12 (50.0%)	17 (70.8%)	50 Gy/25fx	Day 1–14, 22–35	80 mg/m^2^	0 (0%)	-	20 (83.3%)	
Inomata *et al.* [24]	2016	37	5 (13.5%)	27 (73.0%)	45 Gy/25fx	On RT day	80 mg/m^2^	5 (13.5%)	4 (10.8%)	PR/CR: 21 (56.8%)	
Hiratsuka *et al*. [25]	2018										74.70%
Present study	2020	54	23 (42.6%)	33 (61.1%)	50.4 Gy/28fx	Day 1–14, 22–35 on RT day	80 mg/m^2^	10 (18.5%)	7 (13.0%)	44 (81.5%)	73.80%

fx = fractions; PR= partial response; PR/CR = partial response/complete resonse.

There are two options for preoperative RT for LARC: short-course RT with 25 Gy/5 fractions or long-course CRT with 45–50.4 Gy./25–28 fractions. The Polish [5] and Trans-Tasman Radiation Oncology Group (TROG) trials [6] showed no difference in LCR, survival rates or adverse events between short-course RT and long-course CRT. However, the TROG trial included only T3 cases, and therefore, the usefulness of short-course RT in T4 cases is not clear. In fact, the National Comprehensive Cancer Network, European Society for Medical Oncology, and the European Organization for Research and Treatment of Cancer guidelines recommend long-course CRT rather than short-course RT for T4 cases [7–9]. Regarding the irradiation field in long-course CRT, there have been reports on pelvic irradiation using the L4/5 boundary as the upper edge [26], pelvic region irradiation using the L5/S1 boundary as the upper edge [4, 6, 10–12, 27], and local irradiation only in the rectum and pararectal region [3]. Of these, pelvic irradiation with L5/S1 as the upper edge is the most common. For the total dose, there are reports on the use of 45 and 50.4 Gy for long-course CRT in the pelvic region. There are also reports for boost irradiation to the rectal region after pelvic irradiation with up to 45 Gy [14, 28, 29]. In our study, the upper margin was set at L5/S1 (Fig. 1a), and no recurrence was observed in the marginal region of the irradiation field. Considering that there were many T4 cases in our study, it appears that the L5/S1 setting was sufficient. The prescribed dose was 45–46 Gy/23–25 fractions in 8 cases, and 50–50.4 Gy/25–28 fractions in the 46 cases in this study. There were no significant differences in acute and late adverse events between two groups. The results of both acute and late adverse events were comparable to those reported in previous trials. S-1 monotherapy may result in fewer adverse events due to the mild toxicity of this chemotherapeutic agent. Taken together, 50.4 Gy/28 fractions with S-1 alone is considered a well-tolerated treatment. As for local control, the 5-year LCR was 85.9%, which was lower than past results. As mentioned above, the higher number of T4 cases (42.6%) may be one reason for the low LCR. T4 status was the only poor prognostic factor for pCR in this study. Another reason was that not all patients received RT of ≥50 Gy. The 5-year LCR was 89.6% in 46 patients who received RT of ≥50 Gy, which was comparable to previous RCTs mentioned above. Since adverse events were well-tolerated in patients treated with RT of 50–50.4 Gy, preoperative CRT with 50–50.4 Gy/25–28 fractions and S-1 alone seem useful treatment options.

Our study was limited due to its retrospective nature. However, there are only a few reports on preoperative CRT with S-1 alone in patients with LARC, especially concerning its long-term results. To the best of our knowledge, our study included the largest number of preoperative CRT cases treated with S-1 alone (Table 6). Another limitation of this study was heterogeneous treatment. Even though the treatment protocol was not identical, we showed efficacy and tolerability of 50–50.4 Gy RT in combination with S-1 alone by comparing LCR and adverse events between different total dose groups (45–46.8 vs 50–50.4 Gy). Our results showed that preoperative CRT with S-1 alone for patients with LARC could contribute to favorable survival, considering the relatively high proportion of T4 disease patients with mild, acute and late toxicities in our study.

In conclusion, our results show a favorable pCR rate, downstaging rate and survival rate, especially considering the relatively high rate of T4 cases. The incidence of grade 3 acute and late toxicities was low, and the therapeutic regimen seemed well tolerated. Our results suggest that S-1 alone may be a treatment option in preoperative CRT for LARC.
